# Estimating the prevalence of pediatric hematological malignancies in Al-Madinah Al-Munawwarah, Saudi Arabia

**DOI:** 10.15537/smj.2023.44.5.20220915

**Published:** 2023-05

**Authors:** Faisal S. Alhajori, Mohammed H. Makkawi, Sultan Z. Alasmari, Ahmad A. Shaikh, Mirza A. Baig

**Affiliations:** *From the Ministry of Health (Alhajori); from Maternity and Children’s Hospital (Baig), Al-Madinah Al-Munawwara; and from the Department of Clinical Laboratory Sciences (Makkawi, Alasmari, Shaikh), Faculty of Applied Medical Sciences, King Khalid University, Abha, Kingdom of Saudi Arabia.*

**Keywords:** pediatric, hematological malignancies, prevalence, leukemia, lymphoma

## Abstract

**Objectives:**

To provide an updated estimate to the prevalence of pediatric hematological malignancies (HMs) in the Al-Madinah Al-Munawwara, Saudi Arabia.

**Methods:**

This is a retrospective study that was carried out between 2016 and 2022. The study population was comprised of 171 children under 16 who had been diagnosed with HMs. The data was compiled from King Salman Medical City’s Maternity and Children’s Hospital, Al-Madinah Al-Munawwarah, Saudi Arabia.

**Results:**

Among the 171 HM patients (64% males and 36% females), 13 subtypes were identified, with B-cell acute lymphoblastic leukemia having the highest incidence (70.3%). Acute myelomonocytic leukemia (8.7%), T-cell acute lymphoblastic leukemia (4.7%), and acute promyelocytic leukemia (3.5%) were the next most common types of HMs. Other rare cases were also found.

**Conclusion:**

Prevalence rate can be utilized to monitor the progression of disease incidence. Here, HMs demonstrated a pattern of increasing incidence in males over a 7-year period, with a higher rate in early childhood. There were 13 types of HMs diagnosed, with B-acute lymphocytic leukemia having the highest incidence. Although juvenile cancer is rare, it is nonetheless a significant cause of mortality in children. A successful prognosis requires prompt and accurate diagnosis and treatment.

**P**ediatric tumors are rare and the primary cause of death in children with cancer. Tumor types, molecular characteristics, and pathogenesis are all unique and often the result of a single genetic driving event.^1^ The World Health Organization (WHO) classification of pediatric tumors was created in response to special diagnostic challenges associated with childhood malignancies. Morphology, immunohistochemistry (IHC), and molecular characteristics are all at the core of WHO classifications.^1^

A recent report provided an updated estimate of worldwide cancer incidence; it is estimated that the number of new cancer cases reached 19.3 million globally.^2^ In addition, the same report estimated the number of worldwide deaths from cancer reached 10 million in the year 2020.

Childhood cancer incidence is increasing, with the United States reporting a 0.6% rise every year.^3^ Pediatric malignancies are the second highest cause of death in developed countries, and HMs are the most prevalent cancers in children, with leukemia being the most common type.^3,4^ According to the most recent data, low-income countries have the highest pediatric cancer mortality and mortality to incidence ratios. Even though the incidence of childhood cancer has increased over the last decade, overall mortality has decreased.^5^

The prevalence of pediatric leukemia in Saudi Arabia, particularly among the Saudi population, is a source of concern for healthcare providers. According to reports, pediatric leukemia is more common in male than in female children in Saudi Arabia, and it ranks top among children under the age of 14.^6^ It has been additionally reported that the frequency of pediatric leukemia varies throughout Saudi Arabia, with the Al-Madinah Al-Munawwarah region reporting the lowest prevalence of leukemia cases and the central region reporting the highest.^6^ In contrast, in accordance with the regional distribution of childhood leukemia in Saudi Arabia from 1999 to 2008, the overall number of leukemic cases was 2021, with the Al-Madinah Al-Munawwarah region having 142 (7%) cases among all regions, making it more prevalent than some other regions.^7^

The present study aimed to provide an updated estimate to the prevalence of pediatric hematological malignancies in the Al-Madinah Al-Munawwarah region, Kingdom of Saudi Arabia between 2016 and 2022 using data that was compiled from an authoritative database of the Maternity and Children’s Hospital, King Salman Medical City, Al-Madinah Al-Munawwarah, Saudi Arabia.

## Methods

This retrospective study was carried out between 2016 and 2022 in 171 patients diagnosed with HMs. Male and female pediatric patients who had been diagnosed with hematological malignancies were included. The data was compiled from an authoritative database of the Maternity and Children’s Hospital in King Salman Medical City, Al-Madinah Al-Munawwarah, Saudi Arabia. The local policy of the hospital identifies children up to 16 years of age as pediatric. The control group had 50 participants, of which 31 (62%) were male and 19 (38%) were female. The controls ranged in age from 1 year to 16 years, whereas patient ages ranged from 1 week to 16 years.

Patients aged over 16 years old were excluded during data collection. Patients who were missing one of the required variables such as medical registration number, gender, age, and final confirmatory diagnosis results were excluded. Ethical approval (Approval No: 2022-2120) was obtained from the research ethics committee at King Khalid University (HAPO-06-B-001) and the study was carried out in accordance to the principles of Helsinki Declaration.

Data were represented as numbers and percentages. The data are descriptive and the differences between groups were not compared statistically.

## Results

In our study, 171 patients were diagnosed with HMs according to all standard diagnostic criteria. The control group had 50 participants, who had no suspicion of other related diseases. To examine the demographic features of patients, descriptive statistics were performed to calculate frequencies and percentages. Descriptive statistics help to understand, represent, and summarize data in an understandable and comprehensive manner. According to the diagnostic criteria for HMs, 13 types of HMs were observed in this study. Table 1 summarizes the frequencies and percentages of patients with HMs. As is evident, of the HMs cases, the majority were males (64%), and 36% were females. B-cell acute lymphoblastic leukemia (B-ALL) reported the highest rates (70.3%). Out of 121 B-ALL patients, 74 (61.2%) were male, and 47 (38.8%) were female. Meanwhile, the second most common subtype in our findings was acute myelomonocytic leukemia (AML-M4) which comprises 8.7%. Followed by T-cell acute lymphoblastic leukemia (T-ALL) which ranks third (4.7%), acute promyelocytic leukemia (AML-M3) (3.5%), acute monoblastic leukemia (AML-M5) (2.3%), and mixed-phenotype acute leukemia (MPAL) comprises (2.3%). Some other rare cases were noted like myelodysplastic syndrome (MDS) (1.7%), juvenile myelo-monocytic leukemia (JMML) (1.7%), acute myeloid leukemia with maturation (AML-M2) comprises (1.2%), acute erythroid leukemia (AML-M6) (1.2%), and Burkitt’s lymphoma (L3) (1.2%), while prolymphocytic leukemia reported the lowest number with (0.6%).

**Table 1:** - Distribution of HMs cases according to subtypes and gender.

HMs subtypes	Male	Female	Total
B-ALL	74 (61.2)	47 (38.8)	121 (70.3)
AML-M4	8 (53.3)	7 (46.7)	15 (8.7)
T-ALL	8 (100)	0 (0)	8 (4.7)
AML-M3	4 (66.7)	2 (33.3)	6 (3.5)
AML-M5	2 (50.0)	2 (50.0)	4 (2.3)
MPAL	2 (50.0)	2 (50.0)	4 (2.3)
JMML	3 (100)	0 (0)	3 (1.7)
MDS	3 (100)	0 (0)	3 (1.7)
AML-M2	1 (50.0)	1 (50.0)	2 (1.2)
AML-M6	1 (50.0)	1 (50.0)	2 (1.2)
Burkitt’s lymphoma	2 (100)	0 (0)	2 (1.2)
Prolymphocytic leukemia	1 (100)	0 (0)	1 (0.6)
Total	109 (64.0)	62 (36)	171 (100)

Values are presented as number and percentages (%). HMs: hematological malignancies, B-ALL: B-cell acute lymphoblastic leukemia, AML-M4: myelomonocytic leukemia, T-ALL: T-cell acute lymphoblastic leukemia, AML-M3: Acute promyelocytic leukemia, AML-M5: acute monoblastic leukemia, MPAL: mixed-phenotype acute leukemia, JMML: juvenile myelo-monocytic leukemia, MDS: myelodysplastic syndrome, AML-M2: acute myeloid leukemia with maturation, AML-M6: acute erythroid leukemia

To evaluate the distribution of hematological malignancies (HMs) according to subtypes and age, Table 2 was generated. The average age of all patients was 5 years, and the cases of HMs were distributed over 3 age groups. Differences were found in the prevalence of HMs among these 3 age groups (early childhood, middle childhood, and late childhood). Early childhood (≤5 years old) was shown to have the greatest prevalence of HMs (64.9%). While middle childhood (6-12 years old) represented 29.8% and late childhood (13-16 years old) represented 5.3% which constituted the lowest rate of HMs. Interestingly, B-ALL had the highest incidence rate among the 3 groups. Prolymphocytic leukemia, which had the lowest prevalence of all HMs, was diagnosed in early childhood.

**Table 2:** - Distribution of hematological malignancies (HMs) cases according to subtypes and age. Early childhood (≤5 years old), Middle childhood (6–12 years old), and late childhood (13–16 years old).

HMs subtypes	Early childhood	Middle childhood	Late childhood
B-ALL	81 (66.9)	35 (28.9)	5 (4.2)
AML-M4	10 (66.6)	4 (26.7)	1 (6.7)
T-ALL	4 (50.0)	3 (37.5)	1 (12.5)
AML-M3	3 (50.0)	3 (50.0)	0 (0)
AML-M5	3 (75.0)	0 (0)	1 (25.0)
MPAL	1 (25.0)	2 (50.0)	1 (25.0)
JMML	3 (100)	0 (0)	0 (0)
MDS	2 (66.6)	1 (33.3)	0 (0)
AML-M2	1 (50.0)	1 (50)	0 (0)
AML-M6	1 (50.0)	1 (50)	0 (0)
Burkitt’s lymphoma	1 (50.0)	1 (50)	0 (0)
Prolymphocytic leukemia	1 (100)	0 (0)	0 (0)
Total	111 (65.1)	51 (29.8)	9 (5.3)
Values are presented as number and percentages (%). HMs: hematological malignancies, B-ALL: B-cell acute lymphoblastic leukemia, AML-M4: myelomonocytic leukemia, T-ALL: T-cell acute lymphoblastic leukemia, AML-M3: Acute promyelocytic leukemia, AML-M5: acute monoblastic leukemia, MPAL: Mixed-phenotype acute leukemia, JMML: juvenile myelo-monocytic leukemia, MDS: myelodysplastic syndrome, AML-M2: acute myeloid leukemia with maturation, AML-M6: acute erythroid leukemia			

## Discussion

Globally, the incidence of childhood cancer in those under 15 years of age is 10 to 18 per 100,000.^8^ A recent study discovered that the incidence of malignant blood disease is high among children in Saudi Arabia, where one-fourth of the population is under the age of 14, rendering it a critical public health concern.^6^ As in other developing countries, the incidence of cancer in Saudi Arabia has increased over the years despite the development and improvement of health care facilities, diagnostic procedures, and a fast referral system that sends patients to specialized hospitals and oncology centers in major cities for further diagnosis and treatment.^6^

The current study focuses on the estimation of the prevalence of HMs among children aged 0 to 16 years, to assist in the control of the HM cases. Patients were categorized by age based on the principle that children develop in phases involving various biological, emotional, and psychological changes as they progress from birth to the end of childhood. This study utilized 3 age categories: early childhood (0-5 years), middle childhood (6-12 years), and late childhood, also called pre-adolescence (13–16 years).^9^

Our study included 171 HM cases, of which 64% were male and 36% were female; the children ranged from 1 week to 16 years old. All 171 cases were analyzed. Our observations were somewhat consistent with a 2018 study published by Namayandeh et al^10^ which was conducted on children with leukemia aged 0–14 and found a slightly higher incidence in males than in females globally. The reported mean age in our study was 5 years. Broadly, prevalence was high in the early period of childhood and decreased with age, which is consistent with a prior report that early childhood features the highest rate of HMs.^9^

Our observations found that B-ALL is the most diagnosed subtype of HMs in early childhood, with higher incidence in males than in females. This finding agreed with a previous study conducted in Saudi Arabia, where B-ALL was the leukemia type with the highest incidence in children aged <14 years.^6^ The study comprised 8712 cases of leukemia over a period of time and concluded that B-cell leukemia is the most common case in all regions, followed by T-cell leukemia. In addition, a more recent report has evaluated the prevalence of B-ALL among individuals who developed ALL with a mean age of 13.6 years in King Abdulaziz University Hospital, Jeddah, Saudi Arabia.^11^ In comparison to T-ALL, B-ALL had a 51.8% increase in total reported cases, which is consistent with our findings.

Amyelomonocytic leukemia was the second most common type of HMs in the current study, which contradicted the observation of Bashasha et al^12^ that AML-M3 is the more common AML subtype in children <18 years of age.

T-ALL, which is an uncommon but aggressive and life-threatening subtype, ranked third among all cases with an incidence of 4.7%. This result agrees with the above mentioned study carried out in Saudi Arabia, where T-ALL comprised 5.3% of leukemia cases in Saudi children aged under 14 years.^6^

Acute myeloid leukemia or acute promyelocytic leukemia (APL), the rare form of AML, came after T-ALL in terms of prevalence, representing 3.5% of all HM cases and 20.6% of AML cases.^3^ The percentage of APL patients in the United States is similar to that in Central and Northern Europe, accounting for 5-7% of pediatric AML cases.^13^ Using data collected from nine cancer care centers in Saudi Arabia over an eight-year period, it was observed that APL accounts for 14.5% of 207 AML cases in children, which is nearly comparable to our findings.^14^ Nonetheless, despite previous findings, the prevalence of APL in Saudi Arabia remains uncertain.^14^

Other rare subtypes, namely AML-M5 and MPAL, constituted 4.6% of total cases in this study. Acute monocytic leukemia is one of the most common types of AML and is classed as M5 in the French-American-British classification. Patients with AML-M5 have a poor prognosis, with a 3 year survival rate of 26% and overall survival of 31%. Mixed-phenotype acute leukemia is an uncommon type of pediatric HMs that accounts for fewer than 5% of pediatric acute leukemias.^16^ A few other very rare subtypes were noted, such as MDS (1.7%), JMML (1.7%), AML-M2 (1.2%), AML-M6 (1.2%), Burkitt’s lymphoma (1.2%), and prolymphocytic leukemia (0.6%).

A recent study analyzed the epidemiological trend of childhood leukemia in Saudi Arabia and discovered that the Riyadh region has the greatest prevalence of childhood leukemia with 578 (28.6%) cases while the Al-Madinah Al-Munawwarah region ranks fifth with 142 (7%) cases.^7^ By comparing the epidemiological trend of childhood leukemia cases in the Al-Madinah Al-Munawwarah region between 1999 and 2008 to our observations obtained between 2016 and 2022, we found a 16.9% rise in the epidemiological trend of leukemia cases in the region.

The primary objective of this research was to encourage researchers to carry out a study to report the prevalence and incidence of HMs in every region of Saudi Arabia regularly in terms of case management and control, as there is a lack of documented and updated investigations. The study of hematological malignancy prevalence and incidence is necessary, but it is challenging owing to the difficulty of disease diagnosis; therefore, we recommend that researchers be encouraged to report confirmed cases for epidemiological trend considerations.

### Study limitations

The constraint of this study is the different medical records systems used to collect data. Furthermore, follow-up of patients during treatment and determination of survival rates for patients with HMs is valuable. However, in the current study, follow-up of patients during treatment as well as study of survival rate was difficult, as patients receive treatment in other independent centers.

In conclusion, this study on the prevalence of pediatric HMs in the Al-Madinah Al-Munawwarah region could be used as background information to support the investigation of epidemiological trends. Specifically, as prevalence in turn determines the epidemiology of a disease, this study will serve as a reference for subsequent studies that measure disease prevalence in the same region. Over a 7-year period, HMs showed an increased probability of incidence in males more so than in females, and early childhood was the most commonly affected age group. Thirteen types of HMs were diagnosed, with B-lymphocytic leukemia having the highest incidence rate. Although childhood malignancy is rare, it is still a major cause of death in children.
